# Case Report: Facial Malassezia folliculitis following infliximab treatment in Crohn’s disease

**DOI:** 10.3389/fimmu.2025.1611893

**Published:** 2025-08-12

**Authors:** Liji Chen, Yanyan Ma, Shaoyu Cheng, Beiping Zhang, Tianwen Liu, Xiying Zhao

**Affiliations:** ^1^ The Second Clinical Medical College of Guangzhou University of Traditional Chinese Medicine, Guangdong, Guangzhou, China; ^2^ Clinical College of Chinese Medicine, Hubei University of Chinese Medicine, Hubei, Wuhan, China; ^3^ Department of Gastroenterology, The Second Affiliated Hospital of Guangzhou University of Traditional Chinese Medicine, Guangdong, Guangzhou, China

**Keywords:** inflammatory bowel disease, Crohn’s disease, infliximab, Malassezia folliculitis, adverse event, fungal infection, biologic, tumor necrosis factor-alpha

## Abstract

Infliximab (IFX), a first-line treatment for moderate to severe Crohn’s disease (CD), has immunomodulatory effects that increase the risk of opportunistic infections. Although IFX-associated invasive fungal infections have received widespread attention, IFX-associated superficial cutaneous fungal infections, such as Malassezia folliculitis (MF), have not been fully recognized. Herein, we present a case of a 19-year-old female patient with moderately active CD who rapidly developed facial erythema, inflammatory papules, and nodular lesions after treatment with IFX. Initially, she was diagnosed with acne vulgaris, but topical and oral antibiotic treatments were ineffective. After completing five IFX infusions, she not only had no relief of gastrointestinal symptoms, but also had progressive exacerbation of the cutaneous lesions. Fungal microscopy revealed abundant Malassezia spores, confirming the diagnosis of MF. Subsequently, IFX was discontinued, and treatment was switched to Ustekinumab (UST). Following this therapeutic adjustment, the patient demonstrated simultaneous resolution of both gastrointestinal and cutaneous symptoms. Notably, the facial lesions completely resolved after three UST infusions without the use of antifungal drugs. This case is the first report of MF induced by IFX therapy in a CD patient. It highlights that acneiform eruptions emerging during biologic therapy may represent cutaneous fungal manifestations. Early recognition and timely adjustment of treatment regimens are essential to prevent potential systemic fungal infections.

## 1 Introduction

Crohn’s disease (CD) is a chronic transmural inflammatory disease that may involve any segment of the gastrointestinal tract (1). Infliximab (IFX), an anti-tumor necrosis factor-alpha (TNF-α) biologic, has emerged as first-line therapy for moderate-to-severe CD by effectively targeting and inhibiting this pro-inflammatory cytokine and improving clinical outcomes (2, 3). However, its immunomodulatory effects may impair host immune defenses, increasing the risk of opportunistic infections (4). Clinical studies have reported that some patients treated with anti-TNF-α biologics develop invasive fungal infections within 6 months, mainly in the gastrointestinal and respiratory tracts (5, 6).

Although invasive fungal infections associated with anti-TNF-α biologics have garnered significant attention, superficial cutaneous fungal infections remain underrecognized. Malassezia species, which are part of the commensal skin microbiota, can be transformed into a pathogenic organism under immunosuppressive conditions, leading to Malassezia folliculitis (MF) (7). MF typically manifests as acneiform lesions. Current clinical reports primarily attribute acneiform eruptions during anti-TNF-α treatment to drug-related effects, while microbiologically confirmed fungal etiologies are seldom described (8, 9).

Herein, we report a unique case of facial MF complicating IFX treatment of CD. This case provides critical insights into the early identification and risk management of opportunistic infections during IFX treatment.

## 2 Case report

**Figure 1:**
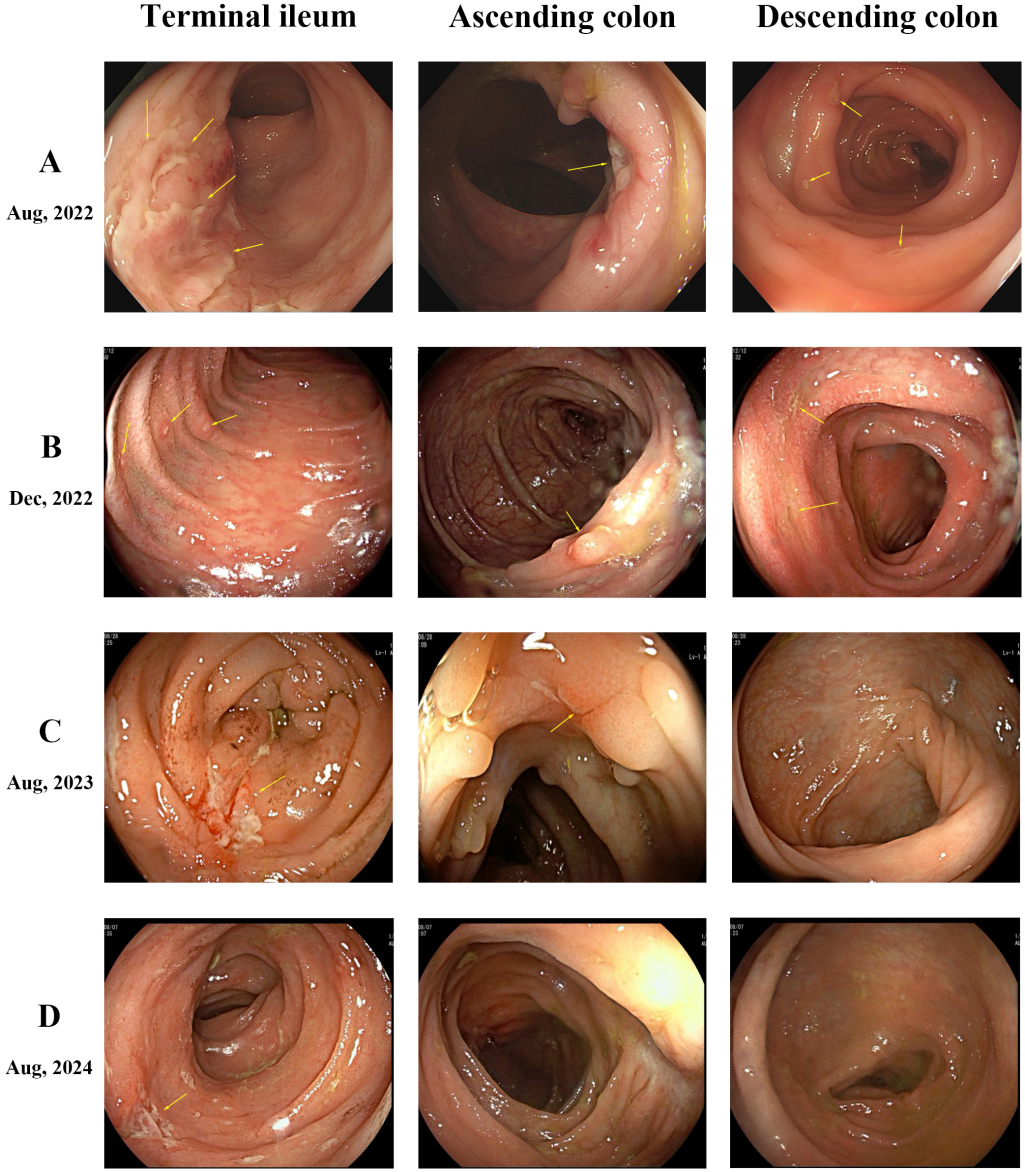
Endoscopic findings. **(A)** Multiple segmental ulcers in the intestine, exhibiting irregular or pinpoint shapes with overlying white moss. **(B)** Persistence of multiple ulcers across intestinal segments, showing no significant overall improvement compared to prior findings. **(C)** Healing of multiple ulcers. The ascending colon displays several white depressed scars and polypoid hyperplasia, while the descending colon mucosa appears normal. **(D)** Normal mucosal appearance in both the ascending and descending colon.

A 19-year-old female was hospitalized with recurrent abdominal pain, diarrhea, and hematochezia. Laboratory tests showed white blood cell count 7.79×10^9^/L (reference range: 3.50-9.50), erythrocyte sedimentation rate 44 mm/h (reference range: 0-32), and C-reactive protein 33 mg/L (reference range: 0-6). Gastrointestinal endoscopy revealed multiple segmental ulcers involving the gastric body, jejunum, terminal ileum, and colonic segments from the ileocecal valve to the descending colon (**Figure 1A**). The ulcer surfaces were covered with white moss, and the interulcer mucosa was normal. Histopathologic examination demonstrated severe infiltration of chronic inflammatory cells in the lamina propria with granuloma formation. CT enterography showed multifocal bowel wall thickening in the terminal ileum, ileocecum, ascending colon, descending colon, and sigmoid colon, the thickest of which was about 1.2 cm. The intestinal mucosa was unevenly strengthened, and the mesenteric blood vessels showed the “comb sign”. Pelvic MRI identified a simple anal fistula. Based on these findings, the patient was diagnosed with moderate active Crohn’s disease with Montreal classification: A2, L3+L4, B1p, and Simplified Endoscopic Score for Crohn’s Disease (SES-CD) score: 17.

The patient initiated standard IFX therapy at 200 mg. Following the first infusion, the patient developed multiple scattered 2–3 mm erythematous plaques, inflammatory papules, and nodular lesions emerged on her face, accompanied by pruritus, with no comedones present. Notably, the patient had no prior history of acne. With increasing doses, these cutaneous manifestations progressively worsened. A dermatologist initially diagnosed acne vulgaris, but topical and oral antibiotic therapies proved ineffective. After five IFX infusions, fungal microscopy with 10% potassium hydroxide (KOH) preparation revealed abundant Malassezia spores. Considering the clinical features and unsuccessful antibiotic treatment history, the diagnosis was revised to MF. Notably, gastrointestinal symptoms persisted during this period, with repeat endoscopy showing an unchanged SES-CD score of 16 (**Figure 1B**), demonstrating failure to achieve both clinical response and endoscopic response targets.

**Figure 2:**
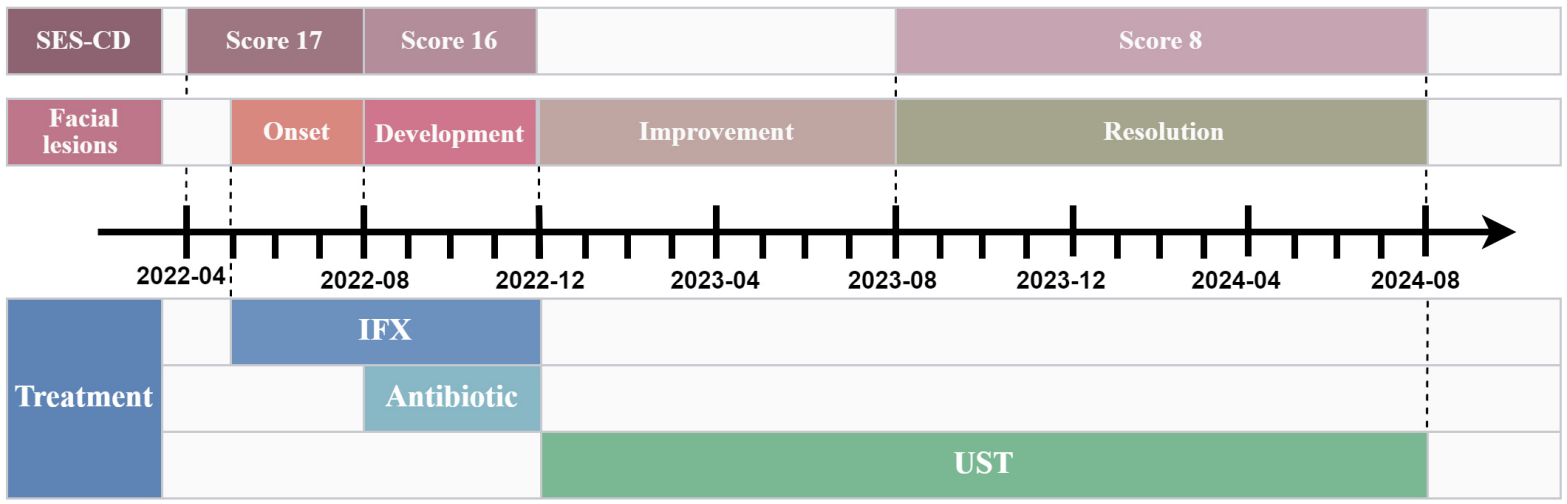
The timeline of disease progression and therapeutic interventions. SES-CD, Simplified endoscopic score for crohn’s disease; IFX, Infliximab; UST, Ustekinumab.

Considering the failure of IFX to induce remission, treatment was adjusted to Ustekinumab (UST) with 260 mg intravenous induction followed by 90 mg subcutaneous maintenance every 8 weeks. Dramatic improvement was observed. Abdominal symptoms markedly resolved after the first UST infusion, and facial lesions began regressing. After 3 courses of treatment, the skin lesions completely disappeared, and endoscopic review showed that the colonic mucosal ulcer was better than before with a SES-CD score reduced to 8 (**Figure 1C**). During this period, the patient did not use antifungal drugs. The patient has remained on UST therapy since then, with colonoscopy after one year demonstrating improvement in ascending colonic mucosal lesions (**Figure 1D**). **Figure 2** outlines the timeline of disease progression and therapeutic interventions.

## 3 Discussion

This is the first case to report the occurrence of facial MF during IFX treatment of CD. The temporal sequence between IFX initiation, cutaneous manifestation onset, IFX discontinuation, and subsequent clinical remission strongly supports a causal relationship between IFX and facial MF. Notably, both gastrointestinal and cutaneous lesions resolved spontaneously after switching to UST without antifungal intervention, suggesting the central role of IFX in this adverse event.

In terms of pathogenesis, IFX-mediated inhibition of the TNF-α signaling pathway may contribute to MF development through a multidimensional mechanism. MF is an opportunistic fungal infection caused by Malassezia species. It typically develops under the influence of interfering factors such as antibiotics, corticosteroids, or immunosuppressive agents (7). TNF-α is not only a central cytokine of pro-inflammatory responses, but also a key factor in antifungal immunity (10–12). It constructs a multilayered antifungal immune defense system through various mechanisms. These mechanisms involve the activation of innate immune cells such as macrophages, neutrophils, and dendritic cells, the regulation of Th1/Th17 differentiation, and the direct inhibition of fungal virulence (13–16). Anti-TNF biologics disrupt the above protective mechanisms, creating an ecological condition for Malassezia pathogenicity.

The diagnostic process in this case highlights the challenges posed by inadequate clinical cognition. MF typically presents as itchy, monomorphic papulopustular skin lesions commonly in areas with abundant sebaceous glands, closely resembling acne vulgaris (17). Given the high prevalence of CD in the adolescent population and the high prevalence of acne vulgaris in this age group, clinicians are highly susceptible to misdiagnosing it as acne vulgaris. Furthermore, drug-induced acne is also similar to MF. It is usually caused by corticosteroids or JAK inhibitors in IBD therapy, but usually involves non-seborrheic areas and responds to antibiotics (18). **Table 1** outlines the key diagnostic differentiating features among these conditions. In this case, the patient’s clinical manifestations, the failure of antibiotic treatment, along with positive fungal microscopy, provides key evidence for differential diagnosis. The limited reporting of acneiform reactions associated with anti-TNF biologics likely contributes to the underrecognition of these cutaneous adverse events in clinical practice (8, 9).

**Table 1:** Key diagnostic features of acne vulgaris, drug-induced acne, and Malassezia Folliculitis.

Feature	Acne Vulgaris	Drug-induced acne	Malassezia Folliculitis
Lesion Characteristics	Comedones, papules, pustules, nodules, and cysts	Erythematous papules or pustules, without comedones	Erythematous papules or pustules, without comedones
Associated Symptoms	Mild or no pruritus	No pruritus	Pruritus
Distribution	Seborrheic areas (face, chest, back)	Non-seborrheic areas (limbs, buttocks)	Seborrheic areas (face, chest, back)
Precipitating Factors	No specific exposure; associated with hormones and bacteria	Specific medication use (corticosteroids, androgens)	Immunosuppression or antibiotic use
Treatment Response	Responsive to antibiotics	Requires drug discontinuation; may respond to antibiotics	Responsive to antifungal therapy

Though MF itself is rarely life-threatening, its clinical significance should not be underestimated. Firstly, acneiform eruptions significantly impact facial aesthetics, potentially exacerbating psychosocial distress, reducing treatment adherence, and ultimately impeding disease management (19–22). More importantly, the rapid progression of MF suggests a state of systemic immune and local microecological imbalance, which may serve as an early warning sign of more serious invasive fungal infections. Meta-analysis data confirm that anti-TNF biologics recipients face increased risk of fungal infections within six months, including life-threatening conditions like gastrointestinal candidiasis, histoplasmosis, and Pneumocystis jirovecii pneumonia (5, 23, 24). Thus, although MF itself is rarely a fatal risk, its occurrence may signal that the patient is already in a state of immune imbalance and needs to be alert to synergistic infections with other opportunistic pathogens.

This case provides insight into biologic selection strategies in CD management. Although IFX is widely used in clinical practice due to its rapid induction of clinical remission, it carries a higher risk of infection compared to other biologics (25, 26). A nationwide study in Korea showed a higher risk of serious infections with anti-TNF-α biologics compared to non-anti-TNF biologics, such as vedolizumab and UST (27). As an IL-12/23 inhibitor, UST specifically blocks the shared p40 subunit of IL-12 and IL-23, preventing their binding to the IL-12Rβ1 receptor. This action inhibits the differentiation of downstream Th1 and Th17 cells and reduces the production of pro-inflammatory cytokines (28, 29). Compared to TNF-α inhibitors, this immunomodulatory mechanism offers greater target specificity and a lower risk of broad immunosuppression. Multiple large-scale cohort studies have demonstrated that UST treatment for CD is associated with a lower risk of serious infections compared to anti-TNF agents (30, 31). It is supported by the simultaneous symptomatic resolution achieved after conversion to UST in the present case.

These observations serve as a cautionary note for risk management in biologic therapy for CD. When prescribing biologics for the treatment of CD, particularly anti-TNF-α biologics, clinicians should maintain heightened surveillance for cutaneous manifestations, particularly in areas with abundant sebaceous glands. Upon identification of adverse reactions, clinicians should promptly adjust the treatment regimen to prevent potential systemic fungal infections.

In conclusion, this case is the first report of IFX-induced facial MF in a CD patient. It highlights the potential risk of anti-TNF-α biologics causing a state of systemic immune and local microecological imbalance, as well as the importance of timely recognition of acneiform eruptions and weighing the risk-benefit ratio of biologics, which provides new insights for early warning of opportunistic infections associated with IBD treatment.

## Data Availability

The original contributions presented in the study are included in the article/Supplementary Material. Further inquiries can be directed to the corresponding author.
